# A simple tourniquet technique for bleeding control after percutaneous hemodialysis fistula and graft interventions

**DOI:** 10.1186/s12882-020-01784-y

**Published:** 2020-03-31

**Authors:** Qiquan Lai, Hui Zhang, Bo Chen, Xuejing Gao, Ling Chen, Bo Tu, Baifei Li, Bo Hu, Fan He, Yong Xu, Ziming Wan

**Affiliations:** 1 Department of Nephrology, The First Affiliated Hospital of Chongqing Medical University, Chongqing, China; 2 Medical Department, The First Affiliated Hospital of Chongqing Medical University, Chongqing, China; 3 Department of Ultrasonography, The First Affiliated Hospital of Chongqing Medical University, Chongqing, China; 4 Department of Nephrology, The First Affiliated Hospital of Jinan University, Guangzhou, Guangdong China; 5 Department of Nephrology, Tongji Hospital, Tongji Medical College, Huazhong University of Science and Technology, Wuhan, Hubei China; 6 Department of Nephrology, The Third Xiangya Hospital of Central South University, Changsha, Hunan China

**Keywords:** Percutaneous intervention, Hemostasis, Tourniquet

## Abstract

**Background:**

The purse-string suture has been widely used for bleeding control after percutaneous interventions through arteriovenous fistula (AVF) and graft (AVG), and it requires suture removal the next day. This study aimed to introduce a simple method using a tourniquet to facilitate hemostasis following AVF or AVG sheath removal after percutaneous procedures.

**Methods:**

Data were retrospectively collected and included all the consecutive patients who received bleeding control with a tourniquet after percutaneous AVF or AVG interventions. Hemostasis was facilitated using the tourniquet technique after sheath removal.

**Results:**

A total of 1966 patients who received the tourniquet technique for bleeding control after percutaneous AVF or AVG interventions were included. Bleeding control was successfully achieved in all patients. Regarding complications, hematoma, thrombosis, and rebleeding occurred in 57 (2.9%), 11 (0.6%), and 8 (0.4%) patients, respectively. Neither pseudoaneurysm nor infection occurred in the patients. Age, gender, pre-existing diseases (including diabetes and hypertension), procedure count, sheath size, hemodialysis access type, and canalization route were similar between patients with and without complications. The primary patency rates at 6,12, 24, and 36 months were 85.0, 64.6, 53.8, and 41.6%, respectively.

**Conclusions:**

The tourniquet technique is an effective and safe approach for facilitating hemostasis after catheter-based percutaneous interventions of hemodialysis accesses.

## Background

Over 750 million persons is affected by chronic kidney disease worldwide, and more than two million patients with end-stage renal disease (ESRD) are dependent on hemodialysis [1, 2]. With the rising requirement of hemodialysis for ESRD, the creation of hemodialysis accesses, including arteriovenous fistula (AVF) and arteriovenous graft (AVG), has become the most common vascular surgery [3]. The greatest challenge of continuing hemodialysis is maintaining patency of hemodialysis accesses as less than half of all accesses remain patent for 3 years [3]. Catheter-based interventions have replaced surgical procedure and are successful in restoring flow in most narrowed and thrombosed hemodialysis accesses [3]. Percutaneous transluminal angioplasty of diseased hemodialysis requires puncture and sheath placement in AVF or AVG. In addition, hemostasis accesses are also punctured and canalized for central venography, angioplasty, and stent placement.

Hemostasis of hemodialysis accesses after sheath removal following catheter-based interventions is a time-consuming process. The hemostasis process may complicate with acute thrombosis, hematoma, and rebleeding. In addition, uremic toxins in patients with ESRD make bleeding control of hemodialysis accesses more challenging following catheter-based interventions. There are only few procedures that were developed to facilitate bleeding control of canalized AVF and AVG. A circular suture was first reported in 1997 by Vorwerk and colleagues, and the next year a similar purse-string suture technique was introduced and has been used as a main measure to facilitate hemostasis of AVF and AVG after percutaneous procedures in many centers for decades [4–7]. The purse-string suture technique was reported to be effective and safe in achieving immediate hemostasis in 2018 [8]. These methods require suture and suture removal the next day after the procedure.

From January 2016, we have been applying a simple technique using a tourniquet without suture to enable effective and safe bleeding control after percutaneous procedures. This study aimed to report efficacy and safety of the tourniquet technique for facilitating hemostasis of AVF and AVG after catheter-based interventions.

## Methods

### Study population

This was a single center retrospective cohort study. Between January 2016 and July 2019, a total of 1966 consecutive hemodialysis patients who underwent catheter-based interventions of AVF or AVG were enrolled. All patients received bleeding control with the tourniquet technique after percutaneous intervention in our center. Patients with coagulation disorders were excluded. Informed consent was obtained from all patients. Demographics, history of underlying renal disease, and procedural records were collected. The Institutional Review Board of the First Affiliated Hospital of Chongqing Medical University reviewed and approved the study protocol.

### Tourniquet technique

**Fig. 1:**
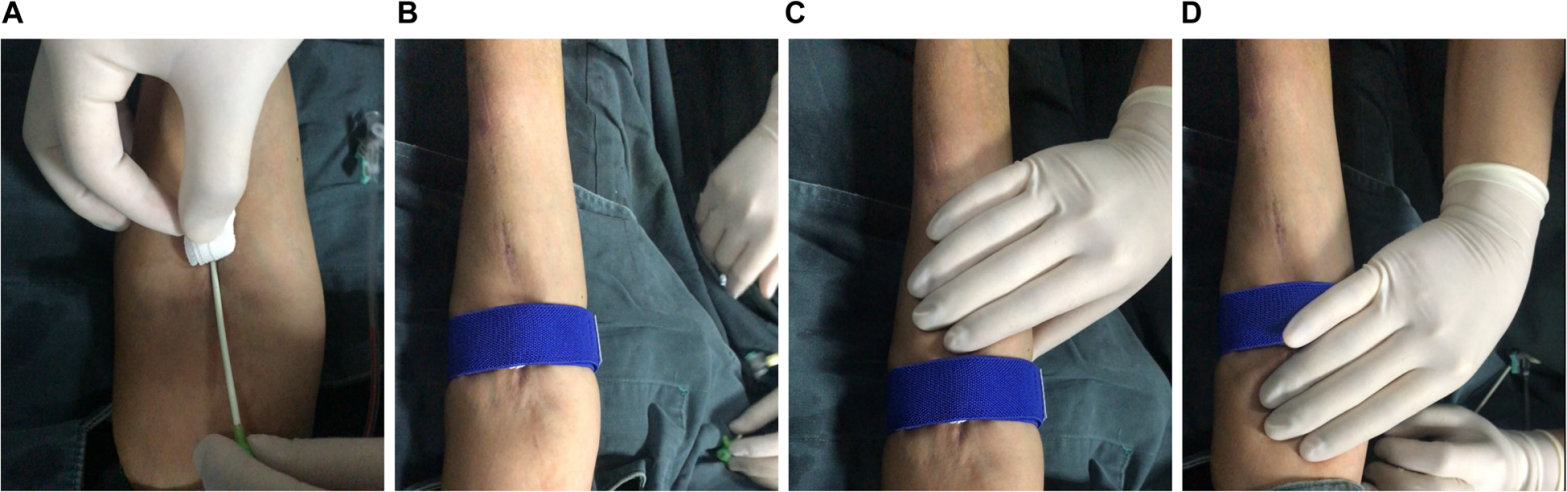
The tourniquet technique. **a** Sheath removal from the hemodialysis access. **b** Tourniquet placement. **c** and **d** Palpating thrills over the hemodialysis access

Percutaneous procedures were performed under local anesthesia. Heparin was administered during all interventions with the same dose of 3125 IU. Sheathes from a size of 5Fr to 7Fr were used via AVF or AVG. At the end of an interventional procedure, absorbent gauze was applied with slight pressure on the puncture site and the sheath was removed. Then, a tourniquet was placed on the absorbent gauze around the upper extremity. The tourniquet was adjusted to prevent bleeding and avoid blocking AVF/AVG blood flow, the latter was checked by palpating thrills over the hemodialysis access (Fig. 1a-d). Twenty minutes later, the tourniquet was removed. Hematoma, thrombosis, rebleeding, pseudoaneurysmal formation, and infection events were recorded in 24 h after the procedure. Anticoagulants or antiplatelet medications were not routinely prescribed after the procedure.

### Follow-up assessments

Follow-up vascular ultrasound examinations were scheduled every 3 months to detect access stenosis, which was a routine practice in our Department. The follow-up periods ranged from 6 to 36 months. Primary patency was defined as the interval from the time of the repair surgery to any intervention designed to maintain the patency of the access.

### Statistical analyses

Data are presented as mean and standard deviation for continuous variables and as number and percentage of patients for categoric variables. Statistical analysis was performed with SSPS version 21.0 software (IBM Corp., Armonk, NY, USA). Comparing means of continuous variables between two groups were performed using the student *t*-test, and categorical variables were analyzed using Fisher’s exact test. Differences were considered significant at a probability level of *P* < .05.

## Results

A total of 1966 patients received the tourniquet technique for bleeding control after catheter-based interventions through AVF or AVG were included. The average age was 59 ± 14 years, and male percentage was 50.8% (Table 1). The underlying renal diseases include glomerulonephritis, diabetic nephropathy, hypertensive nephrosclerosis, polycystic kidney disease, obstructive nephropathy, vasculitis, and others (Table 1). Patients who had underwent 1–3, 4–6, or more than 6 percutaneous interventions accounted for 93.8, 5.7, and 0.5%, respectively (Table 2). The sizes of sheathes we used were 5 Fr (0.2%), 6 Fr (99.6%), and 7 Fr (0.3%) (Table 2). Canalization was performed through AVF (93.4%) or AVG (6.6%) via the arterial (20.2%) or venous (79.8%) segment (Table 2).

**Table 1:** Patient characteristics

Variables	n (%) or median (range)
Number	1966 (100%)
Age, years	60 (22–91)
Male	999 (50.8%)
Underlying renal disease	
Glomerulonephritis	652 (33.2%)
Diabetic nephropathy	508 (25.8%)
Hypertensive nephrosclerosis	334 (17.0%)
Polycystic kidney disease	83 (4.2%)
Obstructive nephropathy	57 (2.9%)
Vasculitis	45 (2.3%)
Others or unknown	287 (14.6%)

**Table 2:** Procedure characteristics

Variables	n (%)
Procedure count	
1–3	1844 (93.8%)
4–6	113 (5.7%)
> 6	9 (0.5%)
Sheath size	
5 Fr	3 (0.2%)
6 Fr	1958 (99.6%)
7 Fr	5 (0.3%)
Access type	
AVF	1836 (93.4%)
AVG	130 (6.6%)
Procedure route	
Venous	1568 (79.8%)
Arterial	398 (20.2%)

Hematoma, thrombosis, and rebleeding occurred in 57 (2.9%), 11 (0.6%), and 8 (0.4%) patients, respectively (Table 3). All complications were well managed without causing severe consequences. Neither pseudoaneurysm nor infection occurred in the patients. We tried to find out possible contributors to the recorded minor complications. However, age, gender, pre-existing diseases (including diabetes and hypertension), procedure count, sheath size, hemodialysis access type, and canalization route were similar between patients with and without complications (Table 4). The primary patency rates at 6,12, 24, and 36 months were 85.0, 64.6, 53.8, and 41.6%, respectively.

**Table 3:** Complications

Variables	n (%)
Hematoma	57 (2.9%)
Thrombosis	11 (0.6%)
Rebleeding	8 (0.4%)

**Table 4:** Characteristics of complications

Variables	Without complications	With complications
Cases	1890 (96.1%)	76 (3.9%)
Age, years	58.0 ± 12.6	58.6 ± 13.9
Male	959 (50.7%)	40 (52.6%)
Diabetes	491 (26.0%)	17 (22.4%)
Hypertension	315 (16.7%)	19 (25.0%)
Procedure count	1.6 ± 1.0	1.5 ± 0.9
Sheath size, Fr	6.0 ± 0.1	6.0 ± 0.0
AVF	1764 (93.3%)	72 (94.7%)
Venous route	1508 (79.8%)	63 (82.9%)

*AVF* Arteriovenous fistula

## Discussion

In this retrospective cohort study, 1966 patients were treated with the tourniquet technique to facilitate bleeding control after catheter-based interventions via AVF or AVG. Only 76 (3.9%) patients had minor complications. The tourniquet technique is effective and safe for bleeding control after percutaneous interventions of hemodialysis accesses.

Manual compression was originally used for bleeding control after percutaneous interventions for narrowed or occluded hemodialysis accesses. Suture-based techniques were then introduced to replace the time-consuming manual compression technique. In 1997, a circular suture, the prototype of the purse-string suture, was first reported as a simple trick to facilitate hemostasis after percutaneous AVF/AVG interventions [4]. In 1998, the purse-string suture technique was formally introduced, and the efficacy and safety of this technique in 20 patients were reported [5]. The purse-string suture technique has thereafter been using to facilitate bleeding control after catheter-based interventions via AVF or AVG in many centers for a long time. Modified purse-string suture techniques, including purse-string sutures with a miniature tourniquet, the loop-suture technique and the Woggle technique, were reported [6, 7, 9]. However, all of those reports only enrolled a small number of patients or procedures. Although the purse-string suture technique is relatively safe [10], an obvious limitation of the purse-string suture is that it requires suture and suture removal on the second day. The tourniquet technique, which we introduced in the present study, is easier to perform than any other suture-based techniques, and no suture removal is needed. The tourniquet technique we used for hemostasis after catheter-based interventions in the present study is the same as the method used for post-hemodialysis puncture site bleeding control. In addition, we believe the tourniquet technique is cost-effective and likely to be used in low socioeconomic regions.

Regarding complications, subcutaneous hematoma, pseudoaneurysm, and failed bleeding control, were reported in 5 to 7% of patients who underwent the purse-string suture-based bleeding control [4–6, 8, 9]. In addition, broken suture and suture-induced complications may occur [9]. In the present study, minor complications, including hematoma, thrombosis and rebleeding, were observed in only 3.9% of patients who received the tourniquet technique. Therefore, the tourniquet technique is safe to be used for bleeding control.

A limitation is that the study was retrospective and only had one arm. There was lack of compression in effectiveness and safety between the tourniquet technique and other bleeding control techniques.

## Conclusions

In conclusion, the tourniquet technique is an effective and safe approach for facilitating hemostasis after catheter-based interventions of hemodialysis accesses. The tourniquet technique could be used as an alternative method for bleeding control following percutaneous AVF or AVG interventions.

## Data Availability

The datasets used and/or analyzed during the current study are available from the author Dr. Ziming Wan (E-mail: wanzm@mail.com) on reasonable request.

## Abbreviation

ESRD: End-stage renal disease
AVF: Arteriovenous fistula
AVG: Arteriovenous graft
